# Unraveling Stage-Specific Metabolites in Human Milk and Their Links to Maternal Physiology: Insights from a Mexican Population

**DOI:** 10.3390/nu17213439

**Published:** 2025-10-31

**Authors:** Imelda Cecilia Zarzoza-Mendoza, Maricela Rodríguez-Cruz, María Cristina Carmona-Isunza, Hilda Sánchez-Vidal, José Carlos Páez-Franco, Cristian Emmanuel Luna-Guzmán, Maricela Morales-Marzana, Juan Manuel Domínguez-Salgado, Judith Villa-Morales, Lourdes Barbosa-Cortés

**Affiliations:** 1 Unidad de Investigación Médica en Nutrición, Unidad de Alta Especialidad (UMAE), Hospital de Pediatría, Centro Médico Nacional Siglo XXI, Instituto Mexicano del Seguro Social (IMSS), Ciudad de México 06725, Mexico; ceci.zarzoza@cic.unam.mx (I.C.Z.-M.); maricela.rodriguez.cruz@gmail.com (M.R.-C.); cristian.luna.guzman@gmail.com (C.E.L.-G.); mmmarzana@live.com.mx (M.M.-M.); jmdomin6@outlook.com (J.M.D.-S.); jusyvm@hotmail.com (J.V.-M.); 2 Red de Apoyo a la Investigación, Universidad Nacional Autónoma de México, Instituto Nacional de Ciencias Médicas y Nutrición Salvador Zubirán, Ciudad de Mexico 14080, Mexico; mcristina.carmona@cic.unam.mx (M.C.C.-I.); paez@cic.unam.mx (J.C.P.-F.); 3 Instituto Nacional de Ciencias Médicas y Nutrición Salvador Zubirán, Ciudad de Mexico 14080, Mexico; hilda.sanchezv@incmnsz.mx

**Keywords:** human milk, metabolomic profile, lactation stages, amino acids, maternal nutritional status

## Abstract

**Background/Objective:** Human milk is an irreplaceable source of nutrition and is essential for the infant’s growth and development right after birth and for early life stage survival. This study aims to characterize and compare the metabolite profiles of colostrum and transitional and mature milk using an untargeted GC-MS approach. Additionally, it explores potential correlations between the identified metabolites and maternal nutritional factors. **Methods:** This was a longitudinal, prospective, and observational study. We included human milk samples from 113 Mexican women who practiced exclusive breastfeeding. Partial least squares-discriminant analysis (PLS-DA) was performed to assess differences among lactation stages. Metabolites showing significant variation across lactation stages were further analyzed using Friedman tests with post hoc Wilcoxon tests and Bonferroni correction. Correlations with maternal anthropometric measures were evaluated. **Results:** Twenty-three metabolites were identified, including amino acids and derivatives, sugars, fatty acids, and energetic metabolites. Alanine and creatinine levels decreased during lactation, while aspartate, serine, and valine levels increased. Rhamnose level was higher in colostrum, whereas decanoic, dodecanoic, and tetradecanoic acid levels increased over time, and that of 11,14-eicosadienoic acid decreased. Lactic acid levels declined across stages. Negative correlations were found between several amino acids and maternal anthropometric variables, while glyceric acid, rhamnose and lactic acid correlated positively. **Conclusions:** Human milk metabolomic profiles display distinct, stage-specific variations shaped by maternal characteristics, reflecting the dynamic physiological and nutritional demands of the developing infant

## 1. Introduction

Human milk is an irreplaceable source of nutrition and is essential for the infant’s growth and development right after birth and for early life stage survival; its composition improves immunological, neurological, and gastrointestinal development and maturation [1,2]. Breastfeeding also provides multiple maternal benefits, including enhanced postpartum recovery, improved metabolic regulation, and long-term protection against type 2 diabetes and breast and ovarian cancers [3,4]. Human milk composition is dynamic and influenced by several factors, including genetics, gestational and infant’s age, circadian rhythm, geographical location, maternal nutrition, and lactation stage [5].

Regarding the lactation stage, colostrum is the first fluid secreted during the first days postpartum (1–7 days), is rich in bioactive factors, such as immunoglobulins, lactoferrin, antibodies, growth factors, and immune cells, and exhibits higher protein content [6]. Transitional milk, produced between 7 and 14 days, represents an intermediate stage in which levels of immune-modulating components decrease while those of energy-yielding nutrients gradually increase, and production ramps up to meet the nutritional needs of the infant [1,7]. Mature milk is secreted from the third week onwards, characterized by a more stable composition, and it provides a balance of nutrients, including fats, proteins, carbohydrates, vitamins, and minerals, to support the infant’s growth and development [1,6].

Previous research has shown that compositional shifts are not limited to macronutrients and bioactive factors but extend to small molecules that comprise the metabolomic profile of human milk. Recent studies focus on mature milk compared to colostrum [8] or transition milk with mature milk [9]. Additionally, another study compared the differences in milk metabolite profile across lactation stages between women with diabetes mellitus and healthy controls [10].

However, new evidence is necessary to identify the human milk metabolite profile at different lactation stages in the Mexican population, because geographical location is a key factor that shapes the human milk metabolome. This study aims to characterize and compare the metabolite profiles of colostrum, transitional and mature milk using an untargeted GC-MS approach. Additionally, it explores potential correlations between the identified metabolites and maternal nutritional factors. The findings contribute to a deeper understanding of human milk composition throughout lactation, supporting optimal infant nutrition.

## 2. Materials and Methods

### 2.1. Research Design

This was a longitudinal, prospective, and observational study to assess whether the metabolite profiles differ between colostrum transition milk and mature milk. This study was performed in accordance with the Declaration of Helsinki, and it was approved by the Research Ethics Committee of the National Commission for Scientific Research of Instituto Mexicano del Seguro Social (IMSS) in Mexico City (Approval # R-2021-785-096). All participants provided written informed consent after the procedures were explained to them.

### 2.2. Study Population

The study included human milk samples from 113 Mexican women who practiced exclusive breastfeeding. Eligible participants were women aged 18 to 35 years who were primiparous, had delivered a single full-term infant (≥37 weeks of gestation), and practiced exclusive breastfeeding during the first month postpartum. Additional inclusion criteria required no history of smoking, alcohol consumption, or substance abuse during pregnancy or lactation. All participants provided written informed consent before enrollment.

### 2.3. Sample Collection and Data Acquisition

Three home visits were scheduled on postpartum days 5–7, 8–15, and 16–28 to collect human milk samples representing distinct lactation stages: colostrum, transition milk, and mature milk, respectively. Not all participants provided samples at all time points. Some missed the first or second visit, while others could not be contacted for the third visit.

Milk expression was performed in the morning (between 8:00 and 9:00 a.m.) for 20–25 min to ensure complete breast emptying. An electric breast pump (Baar, Switzerland, Medela^®^Lactina Select model) was used, with glass collection cups, tubing, and bottles previously sterilized with ethylene oxide.

To facilitate milk ejection, gentle breast massage was applied during expression, which was conducted simultaneously on both breasts. The collected milk was thoroughly homogenized to ensure sample uniformity; an 8 mL aliquot was then transferred into a sterile 15 mL conical tube for subsequent metabolomic analysis, while the remaining milk was returned to the mother for infant feeding. Samples were transported on ice in a container (Costa Mesa, CA, USA, Thermoflask^®^) and stored at −80 °C until further processing.

During each visit, a trained nutritionist administered a standardized questionnaire to collect demographic and anthropometric data, including maternal age, height, pre-pregnancy body weight, and infant sex. At the third visit, maternal body weight and total fat mass were measured using a bioimpedance scale (Arlington Heights, IL, USA, TANITA^®^, BC-585F).

### 2.4. Profile of Human Milk Metabolites

To identify the human milk metabolites profile, an untargeted GC-MS (gas chromatography-mass spectrometry) technique was performed as described by Páez-Franco et al. (2021) [11]. Human milk samples were thawed and centrifuged at 20,000× *g* for 20 min at 4 °C. The fat was removed with a disposable bacteriological loop, and the serum was collected in sterile 1.5 mL tubes. Thirty microliters (30 µL) of human milk serum was derivatized via methoximation followed by the silylation method for subsequent metabolomic analysis in a GC/MS system (Santa Clara, CA, USA, Agilent, 5977A/7890B) with an automatic autosampler (Santa Clara, CA, USA, Agilent, G4513A,) and run under the following conditions: splitless column flow 1 mL/min, inlet temperature 200 °C, electronic ionization (EI) source temperature 200 °C, and interface temperature 250 °C. A column HP5ms (30 m × 250 µm × 0.25 µm, Santa Clara, CA, USA, Agilent) with helium 99.9999% as a mobile phase was employed. The method consisted of a 1 min hold at 60 °C with an increased ramp of 10 °C/min to 325 °C, with a final hold time of 10 min.

The obtained chromatogram files were transformed to .mzdata files using Agilent Chemstation software (Santa Clara, CA, USA, Agilent). Feature detection, spectral deconvolution, and peak alignment were realized using Mzmine2 software (v.54); the parameters used were as follows: retention time range, 5.5–27.5 min; *m*/*z* range, 50–500; *m*/*z* tolerance, 0.5; noise level, 1 × 10^3^; and peak duration range, 0.01–0.2 min. Once the filtered data were obtained, metabolite identification was carried out by comparing the spectral results with the National Institute of Standards and Technology (NIST, v.2.0) spectral library; only results with a probability greater than 70% and a matching score above 700 were considered valid, while values falling below this threshold were labeled as unknown and excluded from the analysis.

### 2.5. Multivariate Statistical Analysis

Initially, a statistical analysis was conducted using a single milk sample per participant, randomly selected from the cohort of 113 women (colostrum, *n* = 42; transition milk, *n* = 36; mature milk, *n* = 34). This approach was used to avoid data pseudo-replication, as not all participants provided samples for all three lactation stages. Then, another analysis was performed on a subset of paired samples from 21 women who provided three milk samples (colostrum, *n* = 21; transition milk, *n* = 21; mature milk, *n* = 21).

Data processing and statistical evaluation were performed using MetaboAnalyst (CAN, v. 6.0) (except when stated otherwise). Missing values were imputed with one-fifth of the minimum positive value detected for each metabolite, based on the assumption that undetected values likely fell below the detection threshold. Repeatability for each metabolite was evaluated using quality control (QC) samples prepared by pooling equal aliquots from all analyzed samples. Metabolites with a relative standard deviation (RSD = SD/mean) greater than 30% in QC samples were excluded, resulting in a final set of 23 metabolites. Data normalization was conducted via sum normalization, followed by log10 transformation and autoscaling (mean-centering and division by the standard deviation).

Partial least squares discriminant analysis (PLS-DA) was applied to enhance class discrimination between lactation stages. For the dataset with paired samples, a multilevel PLS-DA was performed, using subject ID to control for within-subject variation (from the mixOmics package in R). Model performance was assessed through 5-fold cross-validation. The parameters R2 (explained variance) and Q2 (predictive ability) were used to evaluate model quality. Additionally, a 100-permutation test was conducted to validate the robustness of the models. Metabolites contributing most to group discrimination were identified based on variable importance in projection (VIP) scores, with a threshold of VIP ≥ 1.0. For the second dataset with paired samples per participant, the Friedman test using subject as a blocking factor was used to assess differences between lactation stages. Metabolites with *p* < 0.1 were further evaluated using post hoc pairwise Wilcoxon signed-rank tests.

## 3. Results

### 3.1. Demographic Features

One hundred and thirteen human milk samples of women who breastfed exclusively were included in this study. The samples corresponded to colostrum (*n* = 42), transition (*n* = 36), and mature milk (*n* = 35). The main anthropometric and demographic characteristics are shown in Table 1.

### 3.2. Identified Metabolites

Using an untargeted metabolomics approach, 23 different metabolites were found in the human milk serum samples. The identified metabolites belonged to four main groups, amino acids and derivatives (alanine, aspartate, creatinine, glutamic acid, glycine, leucine, proline, serine, threonine, and valine), sugars and derivatives (glyceric acid, glycerol, glycerol phosphate, inositol, and rhamnose), fatty acids and derivatives (11,14-eicosadecanoic acid, decanoic acid, dodecanoic acid, hexadecanoic acid, oleic acid and tetradecanoic acid), and energetic metabolites (alpha-ketoglutarate and lactic acid).

### 3.3. Differences in Metabolite Profiles per Lactation Stage

**Figure 1:**
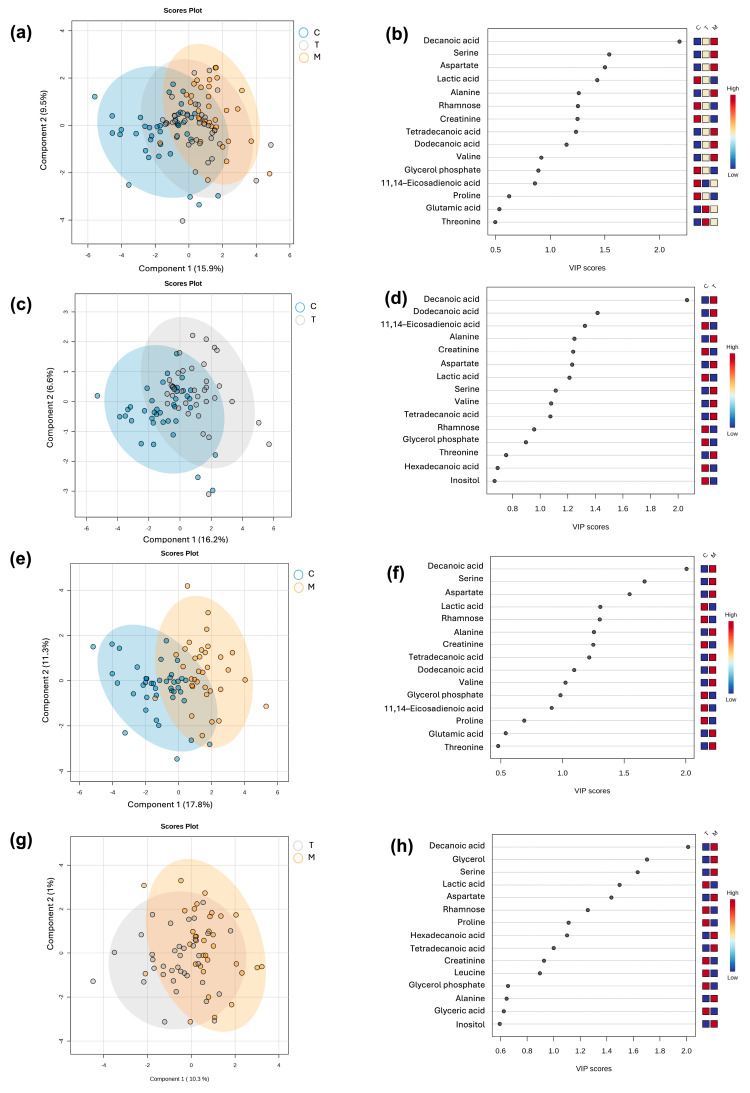
Partial least squares-discriminate analysis (PLS-DA) plots of differential metabolites per milk stage and their corresponding VIP values. C vs. T vs. M (**a**,**b**); C vs. T (**c**,**d**); C vs. M (**e**,**f**); T vs. M (**g**,**h**). C: colostrum; T: transition milk; M: mature milk.

A supervised multivariate partial least squares-discriminant analysis (PLS-DA) was performed to categorize the samples according to the lactation stage. The robustness of the model classification was assessed trough cross validation and permutation analysis for the following comparisons, colostrum versus transition milk versus mature milk (C vs. T vs. M) (Figure 1a); colostrum versus transition milk (C vs. T) (Figure 1c); colostrum versus mature milk (C vs. M) (Figure 1e); and transition milk versus mature milk (T vs. M) (Figure 1g), obtaining adequate prediction parameters, except for the T vs. M group, indicating less variation in the metabolite profile in this type of milk due to the predicted accuracy being less than 0 (Table 2). The variable importance to the projection (VIP) metabolites relevant to each lactation stage is depicted, respectively, in Figure 1b,d,f,h. It was observed that VIP values belonged to fatty acids, amino acids, sugars, and derivatives.

**Figure 2:**
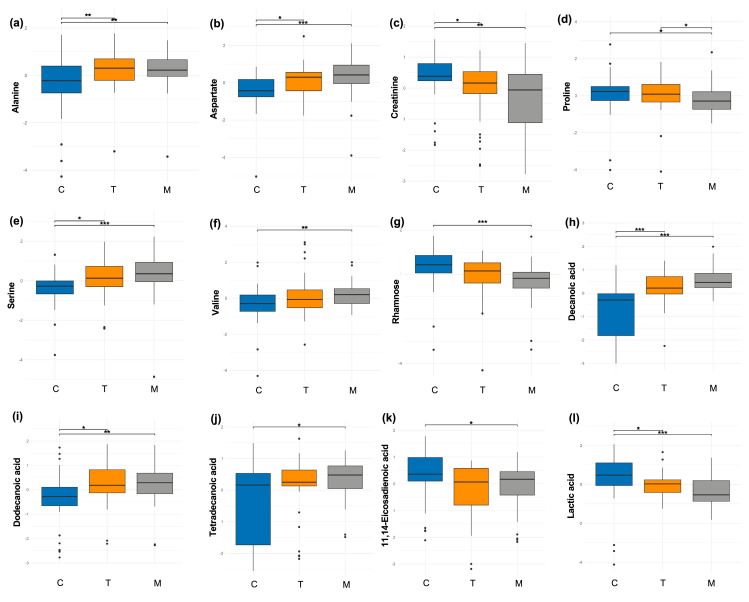
Differences in metabolite profiles by milk stage in all participants (*n* = 113). (**a**) alanine, (**b**) aspartate, (**c**) creatinine, (**d**) proline, (**e**) serine, (**f**) valine, (**g**) rhamnose, (**h**) decanoic acid, (**i**) dodecanoic acid, (**j**) tetradecanoic acid, (**k**) 11,14-eicosadienoic acid, and (**l**) lactic acid. A Friedman test was performed, followed by a post hoc Wilcoxon test with Bonferroni correction. * *p* < 0.05, ** *p* < 0.01, *** *p* < 0.001. C: colostrum; T: transitional milk; M: mature milk.

Then, a comparison analysis was conducted using only one distinct milk sample per woman. Within the class of amino acids and derivatives, alanine levels decreased throughout lactation; its proportion was significantly higher in colostrum compared to both transitional (*p* = 0.002) and mature milk (*p* < 0.001) (Figure 2a). Conversely, aspartate levels increased during lactation, with significantly higher proportions in transitional (*p* = 0.01) and mature milk (*p* < 0.001) compared to colostrum (Figure 2b). Creatinine levels decreased during lactation, with a higher proportion in colostrum relative to transitional (*p* = 0.01) and mature milk (*p* = 0.002) (Figure 2c). Also, proline levels decreased during lactation; the proportion was lower in mature milk compared to colostrum (*p* = 0.01) and transitional milk (*p* = 0.01) (Figure 2d). Serine levels increased during lactation, with significantly higher proportions in transitional (*p* = 0.05) and mature milk (*p* < 0.001) compared to colostrum (Figure 2e). Similarly, valine levels increased in mature milk (*p* = 0.002) compared to colostrum (Figure 2f).

Regarding sugars and derivatives, only rhamnose showed significant differences, with a higher proportion observed in colostrum compared to mature milk (*p* < 0.001) (Figure 2g).

In the class of fatty acids and derivatives, the proportion of decanoic acid increased during lactation, with significantly higher levels in transitional (*p* < 0.001) and mature milk (*p* < 0.001) compared to colostrum (Figure 2h). Similarly, dodecanoic acid levels increased over time, with higher proportions in transitional (*p* = 0.003) and mature milk (*p* = 0.001) than in colostrum (Figure 2i). Tetradecanoic acid showed significantly higher proportions in mature milk (*p* = 0.01) compared to colostrum (Figure 2j). In contrast, the proportion of 11,14-eicosadienoic acid was significantly higher in colostrum compared to mature milk (*p* = 0.01) (Figure 2k).

Finally, in the energetic metabolites class, only lactic acid levels decreased during lactation, showing significant differences in colostrum compared to transitional (*p* = 0.01) and mature milk (*p* < 0.001) (Figure 2l).

**Figure 3:**
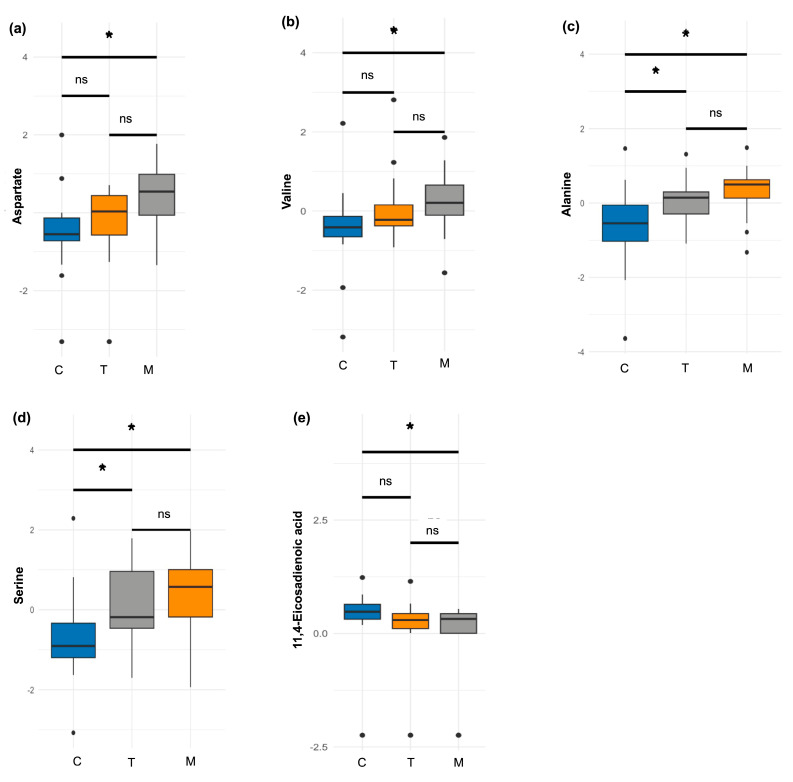
Differences in metabolite profiles by milk stage in the subsample (*n* = 21). (**a**) aspartate, (**b**) valine, (**c**) alanine, (**d**) serine, and (**e**) 11,14-eicosadienoic acid. A Friedman test was performed, followed by a post hoc Wilcoxon test with Bonferroni correction. * *p* < 0.05. C: colostrum; T: transitional milk; M: mature milk; ns: no significative.

A second analysis was conducted on a subsample of women who provided milk samples at all three lactation stages (*n* = 21). Among the amino acids, aspartate (*p* = 0.003) and valine (*p* = 0.004) showed significant increases over time, with higher proportions observed in mature milk compared to colostrum (Figure 3a,b). Additionally, two amino acids exhibited borderline differences. Alanine showed a decreasing trend, with significantly higher proportions in colostrum compared to both transitional (*p* = 0.01) and mature milk (*p* = 0.004) (Figure 3c). Similarly, serine levels decreased during lactation, with higher proportions in colostrum relative to transitional (*p* = 0.04) and mature milk (*p* = 0.002) (Figure 3d).

In the fatty acids and derivatives group, a significant difference was observed only for 11,14-eicosadecadienoic acid, whose levels decreased throughout lactation. Its proportion was significantly higher in colostrum compared to mature milk (*p* = 0.024) (Figure 3e).

In the second analysis, no significant differences were observed in the sugars and derivatives or energetic metabolites classes.

### 3.4. Correlations with Maternal Characteristics

The correlation analysis revealed several moderately significant associations between milk metabolites and maternal anthropometric parameters across lactation stages. Among amino acids, glycine showed negative correlations with maternal body weight at day 7 postpartum (*rho* = −0.23, *p* < 0.05), body fat percentage at day 7 postpartum (*rho* = −0.29, *p* < 0.05), and maternal body weight (*rho* = −0.32, *p* < 0.05) and BMI (*rho* = −0.20, *p* < 0.05) at day 30 postpartum. Glutamic acid also showed negative correlations with pregestational body weight (*rho* = −0.29, *p* < 0.05), maternal body weight (*rho* = −0.28, *p* < 0.05), maternal BMI (*rho* = −0.25, *p* < 0.05) and body fat percentage (*rho* = −0.22, *p* < 0.05) at day 7 postpartum; maternal body weight (*rho* = −0.30, *p* < 0.05), maternal BMI (*rho* = −0.27, *p* < 0.05), and body fat percentage (*rho* = −0.25, *p* < 0.05) at day 15 postpartum; and maternal body weight (*rho* = −0.29, *p* < 0.05), maternal BMI (*rho* = −0.25, *p* < 0.05) and body fat percentage (*rho* = −0.22, *p* < 0.05) at day 30 postpartum. Leucine correlated negatively with pregestational body weight (*rho* = −0.21, *p* < 0.05), maternal body fat percentage (*rho* = −0.23, *p* < 0.05) at day 7 postpartum, and maternal body weight (*rho* = −0.25, *p* < 0.05), maternal BMI (*rho* = −0.21, *p* < 0.05), and body fat percentage (*rho* = −0.22, *p* < 0.05) at day 30 postpartum. Serine showed negative correlations with pregestational body weight (*rho* = −0.22, *p* < 0.05), maternal body weight (*rho* = −0.24, *p* < 0.05), maternal BMI (*rho* = −0.21, *p* < 0.05), and body fat percentage (*rho* = −0.27, *p* < 0.05) at day 7 postpartum; maternal body weight (*rho* = −0.28, *p* < 0.05), maternal BMI (*rho* = −0.24, *p* < 0.05), and body fat percentage (*rho* = −0.22, *p* < 0.05) at day 15 postpartum; maternal body weight (*rho* = −0.28, *p* < 0.05) and maternal BMI (*rho* = −0.25, *p* < 0.05) at day 30 postpartum. Meanwhile, valine correlated positively with maternal body fat percentage at day 30 (*rho* = 0.20, *p* < 0.05).

Within sugars and derivatives, glyceric acid correlated positively with maternal body weight at day 30 postpartum (*rho* = 0.20, *p* < 0.05). Also, rhamnose showed a positive correlation with maternal BMI at day 30 postpartum (*rho* = −0.20, *p* < 0.05).

Regarding fatty acids, tetradecanoic acid showed a moderate positive correlation with BMI at day 15 postpartum (*rho* = −0.20, *p* < 0.05).

**Figure 4:**
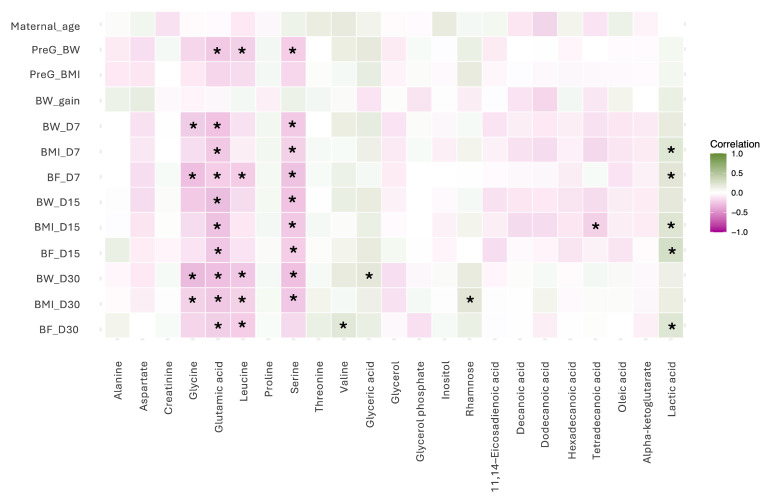
Spearman’s correlation analysis between the identified metabolites and the maternal age and anthropometric variable. * denote the moderate correlations found, with *p* < 0.05. PreG_BW: Pregestational Body Weight; PreG_BMI: Pregestational Body Mass Index; BW_gain: Body weight gain during pregnancy; BW_D7: Body weight at day 7 postpartum; BMI_D7: Body Mass Index at day 7 postpartum; BF_D7: Body Fat % at day 7 postpartum; BW_D15: Body weight at day 15 postpartum; BMI_D15: Body Mass Index at day 15 postpartum; BF_D15: Body Fat % at day 15 postpartum; BW_D30: Body weight at day 30 postpartum; BMI_D30: Body Mass Index at day 30 postpartum; BF_D30: Body Fat % at day 30 postpartum.

In the energy related intermediates group, lactic acid presented positive correlations with maternal BMI (*rho* = 0.21, *p* < 0.05) and body fat percentage (*rho* = 0.21, *p* < 0.05) at day 7 postpartum; maternal BMI (*rho* = 0.22, *p* < 0.05) and body fat percentage (*rho* = 0.27, *p* < 0.05) at day 15 postpartum as well as maternal BMI (*rho* = 0.22, *p* < 0.05) and body fat percentage at day 30 postpartum (*rho* = 0.22, *p* < 0.05) (Figure 4).

## 4. Discussion

This study provides a comprehensive characterization of the dynamic metabolite profile in human milk across distinct lactation stages (colostrum, transitional, and mature milk), using an untargeted GC-MS metabolomics approach. The identified metabolites covered metabolic categories such as amino acids, sugars, fatty acids, and energetic intermediates, highlighting the biochemical complexity of human milk.

The multivariate analysis revealed distinct metabolic signatures associated with the different lactation stages, particularly between colostrum and mature milk, suggesting substantial biochemical modulation during lactation. These changes were predominantly driven by amino acids and fatty acids, which showed the most pronounced variation. Furthermore, several metabolites exhibited significant correlations with maternal anthropometric characteristics, indicating the potential link between maternal physiology and milk composition. Together, these findings highlight the metabolic complexity of human milk and provide insights into how its composition evolves to meet the changing nutritional and developmental needs of the infant.

The dynamic modulation observed in amino acid concentrations throughout lactation aligns with previous reports indicating higher levels of several amino acids in colostrum compared to mature milk [9,12,13,14]. In our study, creatinine and proline showed a marked decline over time. The proline results are consistent with a previous study in which its concentration was higher in colostrum compared to transition or mature milk (*p* < 0.05) [14]. On the other hand, we observed an increase in alanine, aspartate, serine, and valine levels during lactation, suggesting a potential shift toward enhanced metabolic support and tissue synthesis in later stages of infant development [14,15]. It was previously reported that alanine concentration increases through lactation, showing a higher concentration at 3–5 weeks of lactation compared to the 1–2 weeks (*p* < 0.05) [9]. These amino acids are critical for neonatal processes, including nitrogen balance, immune modulation, and protein metabolism, critical during neonatal growth and gut development [9,14,15]. Also, these amino acids in human milk are crucial for infant growth; in previous studies, a negative correlation was found between proline and infant weight at 1 month postpartum [16], serine was positively correlated with weight gain at 6 weeks postpartum [17], and valine was found in lower concentrations in the colostrum of women who delivered infants with intrauterine growth restriction or large for gestational age infants compared to appropriate for gestational age infants [18]. Although causality cannot be inferred from our observational data, these associations suggest a potential role for milk amino acids in supporting early postnatal growth trajectories.

Concerning fatty acids, decanoic, dodecanoic, and tetradecanoic acid levels significantly increased from colostrum to mature milk, a pattern consistent with the increasing lipid requirements of the growing infant [19,20]. This has been reported previously; dodecanoic and tetradecanoic acid levels increase significantly during lactation (R^2^ = 10.0%, *p* < 0.03) [21]. In contrast, 11,14-eicosadienoic acid was more abundant in colostrum; these findings align with those reported in a previous study, in which the concentration was higher in colostrum (*p* < 0.05) compared to transition and mature milk, which may be associated with its putative anti-inflammatory or signaling roles during early postnatal adaptation [22]. These observations highlight the adaptive nature of milk lipid composition in response to infant development needs; the functional roles of these lipids require further exploration.

Lactic acid levels decreased across lactation stages; this may reflect reduced anaerobic metabolism or shifts in microbial activity within the mammary gland or milk itself [23]. Previous studies have reported inconsistent lactate trends between early and later lactation [9], suggesting that multiple maternal or environmental factors, including milk handling and microbial contributions, may influence lactate levels. Given the absence of direct measurements of milk microbiota or mammary metabolic activity, these observations remain hypothesis-generating. Future studies combining metabolomics with microbial profiling and markers of mammary metabolism could clarify these patterns.

The observed moderate correlations between several amino acids and maternal anthropometric variables suggest that maternal nutritional and physiological status may influence the biosynthesis or transport of specific milk metabolites. These findings are consistent with previous reports linking maternal body composition and the metabolite profile observed in human milk [16,24,25,26].

Interestingly, lactic acid levels correlated negatively with some maternal variables, suggesting a possible inverse association with maternal metabolic rate or inflammatory status. These observations highlight the importance of considering maternal phenotype in studies of milk composition and suggest potential avenues for nutritional or lifestyle interventions, though further studies are needed.

The temporal shifts in amino acids and fatty acids may reflect coordinated adaptation to the infant’s changing physiological requirements. The early predominance of amino acids in colostrum may support nitrogen balance, immune development, and gut maturation, whereas the gradual increase in the levels of certain amino acids and medium-chain fatty acids in transitional and mature milk may facilitate tissue growth and energy supply. Although direct functional outcomes were not measured, these compositional changes provide a biochemical rationale for the recognized developmental benefits of human milk. Integrating metabolomics with infant growth and developmental data in future longitudinal studies could help establish mechanistic links between milk composition and infant physiology.

A key strength of this study is the application of untargeted metabolomics on a relatively large and well-characterized cohort, allowing for a detailed temporal resolution of metabolite changes. Additionally, the inclusion of a subsample with longitudinal sampling across all three lactation stages enhances trend interpretation, while standardized sample collection minimized preanalytical variability. However, certain limitations must be acknowledged; some external factors such as maternal diet and genetic variability were not controlled and could influence the milk metabolome. Another limitation is the relatively small sample size in the longitudinal subset, which may reduce statistical power for detecting subtle changes and may introduce bias if participants who provided later-stage samples differ in health behaviors or breastfeeding practices. Future studies integrating dietary assessment, systemic metabolic biomarkers, and multi-omics approaches are essential to fully understand human milk functionality and its role in infant growth development.

## 5. Conclusions

This study provides a detailed characterization of the human milk metabolome across three distinct stages of lactation using an untargeted approach. Significant variations were observed in amino acids, fatty acids, and sugar derivatives over time. The composition of human milk evolved notably from colostrum to mature milk, reflecting the changing physiological and nutritional development of the infant. Additionally, correlations between specific metabolites and maternal anthropometric characteristics suggest that maternal physiology plays a role in shaping milk composition. These findings enhance our understanding of the dynamic nature of human milk and may inform strategies to optimize maternal nutrition and infant feeding practices. As a future perspective, we aim to investigate the impact of maternal diet on the human milk metabolome, as well as the influence of specific metabolites on infant growth.

## Figures and Tables

**Table 1:** Anthropometric and demographic characteristics of the study population.

*n* = 113	
Age (y)	29 (18, 37)
Height (cm)	159.95 ± 5.82
**Body weight (kg)**	
Pre-pregnancy	63 (40, 120)
Postpartum (D30)	63.25 (40, 106)
Body weight gain during pregnancy	10.03 ± 5.04
**BMI (kg/m^2^)**	
Pre-pregnancy	24.01 (18, 43)
Postpartum (D30)	25.05 ± 4.02
**Total body fat mass (%)**	
Postpartum (D30)	32.98 ± 6.86

Values are presented as the median ± DE or median (min, max) depending on the data distribution. D30: day 30.

**Table 2:** Predictability values of partial least squares-discriminate analysis (PLS-DA).

Measure	C vs. T vs. M	C vs. T	C vs. M	T vs. M
R2	0.416	0.331	0.518	0.227
Q2	0.318	0.201	0.422	−0.147
Accuracy	0.576	0.698	0.839	0.614

Q2, R2, and accuracy were calculated via a double cross-validation procedure with 100-fold cross-validation. C: colostrum; T: transition milk; M: mature milk.

## Data Availability

The data are not publicly available due to privacy, legal, or ethical reasons. The data presented in this study are available from the corresponding author upon request.
